# Obscurantism

**Published:** 1892-01-09

**Authors:** 


### PASSING TOPICS.

### "OBSCURANTISM."

The readers of The Hospital who have been at the pains to peruse the several articles on the hospital question, reproduced from the daily and weekly journals, in last week's special supplement, will be in a position to weigh for themselves the sentiments expressed, and to strike the balance. As might have been expected, the balance of opinion will be found largely upon the credit side of the institutional account. It would have been nothing less than startling had the result been other than it is. Where are we to look if not to the Press for an intelligent appreciation of a work of great public utility undertaken by organizations for which there is at present no substitute?

Nothing could have been more unfortunate for the hospitals than that any paper of repute should have followed the lead of the *Pall Mall Gazette*. That journal would seem to have added to its properties a notable and somewhat barbarous word, and like the celebrated Mr. Crummles, who demanded of Nicholas Nickleby a new play in order that a certain pail and mop should be suitably introduced, the autocrat of the *Pall Mall Gazette* could hit upon no better way of utilising his newly acquired "obscurantism" than by employing it in an attack upon hospitals and hospital workers. Happily, the attack has failed; the editor is left with his verbal property on hand, and nothing, so far as we know, has suffered but the reputation of his paper.

Before the *Pall Mall Gazette* takes to writing upon the hospital question again, it would be well if somebody connected with it were at the trouble to make himself acquainted with the necessary details of hospital methods and management. Anything more ridiculously imaginative than the pictures of hospital life presented to its readers has never appeared in a serious and responsible journal. Its attack on "paid officers" is merely vulgar, and may be dismissed with the remark that hospital officers, even if they are remunerated after a fashion, have some settled and useful purpose before them, and thus compare favourably with the mere soldier of fortune, whose pen is ready for either side of a controversy. There is no hospital in London which would not welcome a visit from any fitting representative of the public press, and accord him every courtesy and full information. Publicity is the very life of institutions dependent upon casual support, and if our contemporary thinks otherwise, it is singularly ill informed. But does it?

There is a capricious flavour about some of its articles altogether inconsistent with its business management, and which seems to show that the somewhat spiteful and feminine quality of its writers is not suffered to penetrate to its publishing department. Some cogent reasons why hospitals should advertise in its columns have been lately addressed to the various institutions, and this is a display of obscurantism which may be fairly commented upon. It follows a similar exhibition of abuse and entreaty by that funny little paper of doubtful sex, which seems to have inspired its bigger brother upon this occasion. What are we to believe—the evidence of the leading articles, or the statements in the advertising columns, or is one to be taken as a corrective of the other? Of the two, we should prefer to believe what the hospitals say of themselves. At least they know the truth; the *Pall Mall Gazette* has yet to purge its ignorance.

